# A Medico-Legal Congress

**Published:** 1895-04

**Authors:** Clark Bell

**Affiliations:** New York


### A Medico-Legal Congress.

We have received the following notice:

NEW YORK, March 14, 1895.

There is a project on foot to have a medico-legal congress of the Medico-Legal Society in the summer vacation, either in this city or in some seaside watering place near New York, the point not yet fixed. Would you take an interest in and a part in that congress under either of the branches hereinafter named? The section on Railway Surgery would organize that department of the congress. The Psychological Section, that of psychology, and embrace mental medicine and all questions of social or criminal kind, and appropriate committees on other subjects. It would involve an enrolling fee to cover the cost of it of say $3, so as to pay for a bulletin to include the papers. Would you prepare a paper for it? Would you be willing to attend it? It would be either the end of July or early August, or late August and first of September, and last two or three days. It would embrace—

1. Railway Surgery, under the charge of the officers of that section.

2. Psychological Medicine, under the charge of the officers of that section, embracing all questions of mental medicine.

3. Medico-Legal questions, pure and simple.

4. Miscroscopy, biology, etc.

5. Chemistry and toxicology, etc.
6. Criminology and sociology.

Would you act as one of the officers of the congress?

Faithfully yours,

CLARK BELL.

---

**The quarantine law** has been amended so as to reduce the State health officers's salary to $2500 a year, and all quarantine officers on the coast are cut just half, $150 a month. The bill, as introduced, fixed the salary of coast quarantine officers at $200, in accordance with the governor's wishes; but the "yarb tea" fellows said they ought not to be paid any more than the inspectors on the Rio Grande; that the position is a *sinecure*, and the officers have nothing to do but fish for tarpon, and so the bill stands at this writing, April 8th. Just in what shape it will ultimately come out no one can prophesy. The 24th is a wonderful body of men. There are twenty-two populists in the house, and two in the senate,—a mere sprinkling, and yet they actually shape legislation; they have the democrats by the nose, and lead them to extremes. They are retrenchment-mad. As they get $2 a day they think that amount is enough for anybody. Two days were wasted in saving to the State $200 cut off of the salary of a clerk, and two hours were wasted in cutting the governor's ice allowance from $36 to $18. Could parsimony go further? It is estimated that each hour of legislative session costs the tax payers $200, and yet they will spend days in saving an item of $100.

---

**Is there sound** where there are no ears? In reply to this interrogatory propounded in the March number of the JOURNAL, Dr. C. F. Dight, of New Orleans, sends us an interesting letter. He takes the negative side and says, in effect, that "sound is something requiring for its production a responsive hearing organ. The falling of the tree agitates the air and causes wave-like vibrations to pass through it, which vibrations are not sound, but movement, only, of air, but which, striking the drum membrane of the ear, causes it also to vibrate, and these vibrations are transmitted to the brain, where they give rise to the sensation called sound. Sound, therefore, is the final result of a series of vibrations, beginning usually in the air and ending in the brain, —the sound proper being wholly within the cranium."

We guess the doctor is right, for, it would seem, if sound is, itself, an entity, complete in itself, a deaf man could hear it.

